# Exercise Is Medicine for Nonalcoholic Fatty Liver Disease: Exploration of Putative Mechanisms

**DOI:** 10.3390/nu15112452

**Published:** 2023-05-24

**Authors:** James Westley Heinle, Kara DiJoseph, Angelo Sabag, Sechang Oh, Scot R. Kimball, Shelley Keating, Jonathan G. Stine

**Affiliations:** 1 Division of Gastroenterology and Hepatology, Department of Medicine, Penn State Health Milton S. Hershey Medical Center, Hershey, PA 17033, USA; 2 School of Health Sciences, Faculty of Medicine and Health, The University of Sydney, Sydney, NSW 2006, Australia; 3 Department of Physical Therapy, Faculty of Rehabilitation, R Professional University of Rehabilitation, Tsuchiura 300-0032, Ibaraki, Japan; ohsechang@md.tsukuba.ac.jp; 4 Department of Cellular and Molecular Physiology, College of Medicine, The Pennsylvania State University, Hershey, PA 17033, USA; skimball@pennstatehealth.psu.edu; 5 School of Human Movement and Nutrition Sciences, The University of Queensland, St. Lucia, QLD 4072, Australia; s.keating@uq.edu.au; 6 Department of Public Health Sciences, College of Medicine, The Pennsylvania State University, Hershey, PA 17033, USA; 7 Fatty Liver Program, Penn State Health Milton S. Hershey Medical Center, Hershey, PA 17033, USA; 8 Liver Center, Penn State Health Milton S. Hershey Medical Center, Hershey, PA 17033, USA; 9 Cancer Institute, Penn State Health Milton S. Hershey Medical Center, Hershey, PA 17033, USA

**Keywords:** metabolic-associated fatty liver disease, steatosis, physical activity, cardiorespiratory fitness, biomarker

## Abstract

Exercise remains a key component of nonalcoholic fatty liver disease (NAFLD) treatment. The mechanisms that underpin improvements in NAFLD remain the focus of much exploration in our attempt to better understand how exercise benefits patients with NAFLD. In this review, we summarize the available scientific literature in terms of mechanistic studies which explore the role of exercise training in modulating fatty acid metabolism, reducing hepatic inflammation, and improving liver fibrosis. This review highlights that beyond simple energy expenditure, the activation of key receptors and pathways may influence the degree of NAFLD-related improvements with some pathways being sensitive to exercise type, intensity, and volume. Importantly, each therapeutic target of exercise training in this review is also the focus of previous or ongoing drug development studies in patients with nonalcoholic steatohepatitis (NASH), and even when a regulatory-agency-approved drug comes to market, exercise will likely remain an integral component in the clinical management of patients with NAFLD and NASH.

## 1. Introduction

Upwards of 30% of the world’s population has nonalcoholic fatty liver disease (NAFLD) [1]. At this point in time, there is no regulatory-agency-approved effective drug therapy or cure, and lifestyle modification with dietary change and increased physical activity remains crucial in the clinical management of all types of NAFLD, including nonalcoholic steatohepatitis (NASH), the more severe type. However, most patients with NAFLD do not meet recommended amounts of weekly physical activity [2], and no one optimal diet has been established, although a Mediterranean-informed diet appears to hold the most promise and is recommended by several leading gastroenterology and hepatology societies [3,4]. Even when a regulatory-agency-approved drug therapy becomes widely available, lifestyle modification will always play a key role in the prevention and treatment of NAFLD and lessen the burden of associated extrahepatic disease from cardiovascular disease events and cancer. For this reason, it is important for the benefits of physical activity to be understood. Following years of research, it is widely accepted that regular physical activity and, in particular, exercise training, which is a type of physical activity that is planned, structured, repetitive and with a specific goal in mind [5], leads to many benefits within and outside the liver in patients with NAFLD [6,7]. These benefits include an improvement in liver fat, histologic NASH activity, change in body composition, gain in physical fitness, reduction in markers of cardiovascular risk, improvement in health-related quality of life and possibly a reduction in oncologic risk [7]. Importantly, many of these improvements, including the reduction in magnetic resonance imaging (MRI)-measured liver fat, may occur without clinically significant body weight loss [8].

Despite decades of research in this field, how physical activity and exercise training lead to these benefits remains an area of much interest and ongoing scientific investigation. This interest parallels the ongoing work in drug development, where several pharmacologic agents have progressed to late-phase clinical trials and include drugs with diverse mechanistic targets, including those which impact the Farnesoid X receptor (FXR) signaling [9,10], glucagon-like peptide (GLP)-1 [11], fibroblast growth factor (FGF)-19 [12] and -21 [13], thyroid receptor beta [14], peroxisome proliferator-activated receptor (PPAR) [15] and adenosine monophosphate-activated protein kinase (AMPK) pathways [16]. Moreover, the gut–liver axis, including the gut microbiome, continues to also be of interest in therapeutic clinical trials in patients with NAFLD and NASH [17]. Importantly, exercise training can positively impact these pathways deemed important in NAFLD and NASH drug discovery and may offer a more global approach to this common disease, where multiple pathways can be impacted simultaneously and possibly synergistically or at the very least in concert with these emerging drug therapies. Accordingly, the aim of this narrative review is to synthesize the current literature on the role of exercise training in these mechanistic pathways to underscore and highlight the utility of exercise as a principal management strategy for NAFLD.

## 2. Exercise Training and Mechanistic Pathways Involved in Hepatic Steatosis

### 2.1. AMP-Activated Protein Kinase (AMPK)

AMPK is a fuel-sensing enzyme that is activated by energy stress [18] and is composed of a trimeric complex with a catalytic subunit (α) and two regulatory subunits (β and γ). AMPK plays an important role in global energy balance. AMPK also has a liver-specific role in hepatic de novo lipogenesis, fatty acid oxidation, glycogenolysis and gluconeogenesis. AMPK activity is abnormally low in patients with NAFLD, leading to excessive accumulation of liver fat [19]. For this reason, AMPK remains a drug target of interest, and in fact, the recent Phase 2a STAMP-NAFLD study enrolled 120 patients who were randomized to treatment with PXL770, a direct AMPK activator, or a placebo [16]. Unfortunately, this study did not meet its primary endpoint of statistically significant MRI-determined liver fat reduction; however, subgroup analysis limited only to patients with type 2 diabetes demonstrated a significant reduction in liver fat and corresponding metabolic parameters, including glycemic control.

Animal models of NAFLD demonstrate exercise changes the AMPK pathway, leading to less liver fat accumulation by reducing lipogenesis and increasing fatty acid oxidation [20,21,22,23,24]. Importantly, exercise-induced AMPK activation appears to be dose-related. As exercise intensity and duration increase, ATP usage increases to the point where it cannot be regenerated quickly enough, increasing the AMP/ADP:ATP ratio and activating AMPK [25]. Further, in order to generate additional ATP during exercise, glycogen, which is the main energy substrate used during exercise at higher intensities (typically greater than 70% of VO_2_max), dissociates from AMPK, leading to AMPK activation (glycogen-bound AMPK is inactive) [18,26,27]. Sustained moderate–vigorous intensity exercise seems to be required to deplete glycogen enough to activate AMPK [27,28,29,30]. While several small studies have demonstrated that exercise can favorably impact targets downstream of AMPK, including FGF-21 [31] and also ribosomal protein s6 [32], in patients with NASH, we await a definitive study showing that exercise can directly activate AMPK in this patient population.

There is a clear and consistent body of evidence to support the role of the AMPK pathway as a key pathway modulated by exercise training that appears to be related to both intensity and exercise volume across animal and human studies (Table 1).

### 2.2. Fibroblast Growth Factor (FGF)-19 and -21

FGF is a complex family of peptide hormones that has a crucial implication on regulating energy homeostasis and metabolism [33]. Multiple isoforms of FGF are involved in the cascade, but FGF-19 and FGF-21 are closely related to fat metabolism [34] and are the signaling pathways for both hormones involved in NAFLD and NASH development and the focus of drug discovery [12,35]. Both the FALCON and BALANCED trials investigated the efficacy of FGF-21 analogues pegbelfermin and efruxifermin in patients with NASH [13,35,36].

FGF-19 is expressed in the ileum and is released in response to bile acid stimuli. FGF-19 is activated postprandially, regulating the transcription of hepatic protein and glycogen synthesis and inhibiting hepatic gluconeogenesis. FGF-19 appears to largely act locally on the hepatocytes, whereas FGF-21 tends to be expressed systemically and, of particular interest, in the skeletal muscle, adipose tissue and in the liver tissue [37,38]. Importantly, FGF-21 relies heavily on the coreceptor β-klotho [39]. If there is a lower expression of β-klotho, resistance to FGF-21 has been observed, resulting in impaired fatty acid oxidation [40]. In fact, NAFLD is felt to be an FGF-21-resistant state [41,42].

Because FGF-21 is widely and variably expressed in the human body, it has been challenging to identify the relationship between exercise and FGF-21 expression. Despite this, there is a robust and consistent body of evidence describing this relationship in pre-clinical animal models of NAFLD and in clinical studies involving patients who are inactive, overweight, obese or diabetic [43,44,45]. Importantly, the relationship between exercise and FGF-21 expression appears to exist across differing intensities, where both moderate and vigorous intensity exercise can change FGF-21 expression [46]. In terms of patients with NAFLD, a recent study by Takahashi et al. [47] found that after 12 weeks of resistance training in which participants performed push-ups and squats three times a week on non-consecutive days, serum FGF-21 level was significantly reduced, confirming the results seen with aerobic exercise training in animal models of NAFLD. Furthermore, a study of 24 patients with biopsy-proven NASH reported that 20 weeks of aerobic exercise training significantly reduced serum FGF-21 in parallel with gains in cardiorespiratory fitness [31]. Despite these differences in methodology, it is generally agreed upon that while acute exercise tends to increase plasma levels of FGF-21, perhaps due to increased production by skeletal muscle, chronic exercise training programs of four weeks or more duration lead to a reduction in serum FGF-21 level while simultaneously increasing expression of FGF receptors and β-klotho in not only liver tissue but also in adipose tissue and skeletal muscle [48].

Although closely related to FGF-21, exercise may play a different role in FGF-19 expression. There have been observed correlations between resistance training and upregulation of FGF-19, but further studies are needed to ensure no other external factors are contributing, such as fasting states. These potentially confounding factors may explain the conflicting results of FGF-19 downregulation following an acute one-hour bout of aerobic exercise [49] as well as multiple negative studies, which are limited by small sample sizes. Ramanjaneya et al. [50] found that there was no significant change observed in serum FGF-19 in women with polycystic ovarian syndrome following eight weeks of moderate-intensity aerobic exercise training. Mercer et al. [51] investigated the impact of aerobic exercise training on a small number of women with insulin resistance over a longer 14-week period and found no significant change in serum FGF-19.

To date, the scientific literature suggests that exercise training across different exercise intensities and volumes can activate FGF-21. No conclusions can be made about the relationship between FGF-19 and exercise training.

### 2.3. Glucagon-Like Peptide-1 (GLP-1)

The liver plays a central role in insulin metabolism and is impacted by multiple gut hormones. One such hormone is GLP-1, an incretin, which helps to regulate satiety and lipid metabolism in both the fasting and glucose-stimulated states [52]. GLP-1 has recently emerged at the forefront of drug development in NASH given the recent promising results of early-phase studies using both semaglutide [11] as well as liraglutide [53] and the fact that several GLP-1 receptor agonists are regulatory-agency-approved for the medical treatment of overweight and obesity as well as type two diabetes [54,55].

Exercise can impact serum levels of GLP-1, and in fact, exercise can increase GLP-1 levels in healthy individuals and in persons with obesity and suppress appetite [56,57]. In patients with NAFLD, Kullman et al. [58] measured the impact of a short-term, one-week high-intensity exercise program and found that while GLP-1 level remained unchanged, exercise instead reversed the GLP-1 resistant state of NAFLD by restoring the normal physiologic response of GLP-1 to glucose stimulation. This raises the question of the importance of increasing serum GLP-1 level versus ameliorating GLP-1 resistance, building on previous work showing short-term exercise programs to improve hepatic insulin extraction in patients with NAFLD [59]. To our knowledge, the effects of a long-term exercise training program on GLP-1 have not yet been investigated in patients with NAFLD or NASH.

While GLP-1 and other gut hormones present intriguing avenues of research, the existing scientific data prevent strong conclusions regarding the impact of exercise training programs on this therapeutic target.

### 2.4. Mitochondrial Function and Beta Oxidation

The liver plays a principal role in lipid metabolism as the primary site of de novo lipogenesis and fatty acid oxidation [60]. In fact, lipid-derived energy production in the liver occurs through the β-oxidation of fatty acids [60]. However, mitochondrial defects, which are related to both physical inactivity and obesity [61], reduce the oxidative capacity of the mitochondria, which results in incomplete β-oxidation and the accumulation of metabolic by-products, such as ceramides and diacylglycerides [62]. While intrahepatic triglycerides themselves do not cause hepatic insulin resistance, it is thought that the accumulation of the aforementioned by-products impairs insulin receptor signaling through various mechanisms and has been identified as critical in the pathogenesis of hepatic insulin resistance [63].

Regular exercise has been shown to improve mitochondrial oxidative capacity and increase mitochondrial content, which are related to increases in cardiorespiratory fitness [64,65]. In fact, cardiorespiratory fitness is inversely related to hepatic steatosis [66], and improvements in cardiorespiratory fitness are independently associated with improvements in steatosis [67,68]. As cardiorespiratory fitness has been shown to improve to a similar degree with both high-intensity interval training (HIIT) and more traditional moderate-intensity continuous training [69], this may, in part, explain why HIIT leads to similar improvements in hepatic steatosis to more moderate-intensity continuous exercise despite expending less energy [70].

Although most work in this space has been conducted in animal models, the available data from human studies support the role of improving cardiorespiratory fitness as a key therapeutic target in the management of NAFLD.

### 2.5. Mitochondrial Uncoupling Proteins (UCP)

Mitochondria are vital organelles that are at the forefront of cellular metabolism, especially in the liver, which is the primary metabolic organ in the human body. UCPs are a key component of mitochondrial metabolism and are mitochondrial inner-membrane proteins which mediate proton leak across the inner membrane through anion transport and uncouple substrate oxidation from ATP synthesis [71]. Five key mitochondrial UCPs have been discovered. UCP-1 is found largely in brown adipose tissue and plays a role in thermogenesis and energy expenditure; UCP-2, while fairly ubiquitous, is found in high concentrations in the liver and regulates insulin secretion from pancreatic β-cells as well as fatty acid metabolism; UCP-3 is expressed largely in brown adipose tissue and also skeletal muscle and influences fatty acid metabolism and insulin sensitivity [72,73,74,75]. UCP-4 and UCP-5 are found in the brain. The dysfunction of these transporters has been correlated with various metabolic disorders, such as obesity and diabetes [76,77], and also NAFLD [78,79]. Genetic variation in UCP polymorphisms is also important. In patients with type 2 diabetes, the CC genotype of the UCP-1 rs3811791 polymorphism blunts the response to regular physical activity in terms of insulin resistance and also lipid control, even at guideline-based amounts of 150 min/wk. of moderate-intensity activity [80]. Moreover, the INDOGENIC cohort study demonstrated that even in healthy individuals, the GG genotype for the UCP-2 G-866A polymorphism changed the physiologic response to energy intake, making these individuals more prone to weight gain and overeating over a two year follow-up period [81]. Consequently, targeting UCPs to enhance energy utilization remains an attractive treatment option in NASH. However, when medications with this mechanism of action have been used to induce body weight loss, they have been significantly limited by a strong side effect profile, and this target must be approached with caution [82]. A recent phase 2a trial for a novel mitochondrial uncoupler, HU6, appears to have addressed some of these concerns by demonstrating a favorable side effect profile while inducing significant body weight loss and MRI-measured liver fat reduction in patients with obesity and NASH [83].

Exercise is well known to upregulate the expression of various mitochondrial UCPs. Animal models of aerobic exercise have demonstrated that UCP-1 can be upregulated in brown adipose tissue, white adipose tissue as it browns in response to exercise and skeletal muscle [84,85,86,87]. Animal models have also demonstrated that UCP-2 is modulated by exercise in the vascular endothelium, myocardium, adipose tissue and skeletal muscle [84,88], as is UCP-3 in skeletal muscle [89]. When limited to animal models of NASH, aerobic training reverses dysfunction in UCP-2 in the liver [90]; however, we are unaware of any studies to date in patients with NAFLD or NASH confirming these findings.

In summary, animal models suggest a role for exercise training in upregulating UCPs; however, this remains unexplored in patients with NAFLD and NASH and we await confirmatory data in human subjects to further explore this as a mechanism by which exercise may ameliorate hepatic steatosis.

### 2.6. Peroxisome Proliferator-Activated Receptor (PPAR)-α/γ

PPARα is a nuclear receptor that plays a key role in regulating lipid metabolism. It is specifically activated by fatty acids and their derivatives. The receptor can be found in many key organs, including the liver, and has three subtypes, PPAR-α, PPAR-γ and PPAR-β/δ [91]. When expressed in the liver, PPAR-α is responsible for fatty acid catabolism and energy homeostasis [92,93]. PPAR-γ is heavily involved in glucagon signaling and insulin sensitivity and, thus, is closely related to adiposity-related disorders [94,95]. The PPAR pathway remains one of many targets for drug discovery of anti-steatogenic medications, where late-phase studies have demonstrated PPAR-γ agonists, such as pioglitazone [96], and dual PPAR-α/γ agonists, such as saroglitazar [15], improve insulin resistance and lipid metabolism through regulation of fatty acid metabolism and modulation of inflammatory adipocytokines and adiponectin secretion. Moreover, the PPAR pathway is intricately linked to several other signaling pathways involved in NAFLD and NASH pathogenesis, including FGF-21, AMPK, and uncoupling proteins (UCP) [97,98,99].

The PPAR-α pathway is known to be strongly influenced by exercise training in non-NAFLD populations [100,101,102], including patients who are physically inactive and are overweight or obese [103]. In animal models of NAFLD, multiple studies have shown exercise-induced PPAR-α activation to mediate liver fat reduction [23,95,99,104]. Importantly, the impact of exercise training on the PPAR-α pathway appears to be independent of exercise type, in that PPAR activation has been observed in animals who performed either moderate-intensity aerobic exercise training with either swimming or running [95,99], HIIT [104,105] or resistance training [106]. While exercise per se can influence the regulation of PPAR activity, epigenetic factors may also play a role. For example, maternal exercise during pregnancy may confer protection against the development of NAFLD early in the life of offspring exposed to a high-fat diet. Bae-Gartz et al. [107] demonstrated that male offspring of exercised mouse dams were protected from adult-onset NAFLD mediated through greater activation of PPAR-α. The PPAR-γ receptor may also be affected by exercise training. Batatinha et al. [108] demonstrated that after eight weeks of treadmill training, PPAR-α knockout mice that were fed a high-fat diet to induce NAFLD still experienced a decrease in fat accumulation, perhaps owing to changes in PPAR-γ activity and fatty acid oxidation in the skeletal muscle, which has previously been reported to increase with exercise training in animal models of NAFLD [109,110]. To date, we are unaware of any studies in human subjects with NAFLD which have explored the impact of exercise training on the PPAR pathway to confirm the animal model findings summarized above.

In summary, the current available evidence indicates that exercise-mediated activation of PPAR appears to be mediated by exercise intensity and volume, although the relative contribution of each remains unknown.

### 2.7. Thyroid Receptor (THR)-β

The thyroid gland and NAFLD are intricately linked in that thyroid hormones, including triiodothyronine (T3) and thyroxine (T4), are not only involved in lipid metabolism regulation, glucose uptake and increased size and number of mitochondria [111,112] but also that hypothyroidism is associated with increased NAFLD risk [113,114]. As a result, thyroid-hormone-based treatments, including resmetirom [14], are an attractive therapeutic target in patients with NAFLD and NASH. Two THR subtypes are found in the human body: THR-α, which is predominantly expressed in cardiac tissue, and THR-β, which is most commonly found in the liver and is responsible for the intrahepatic response to T3 [115]. THR-β is a critical receptor in the regulation of cholesterol metabolism and fatty acid oxidation, as exhibited in mouse models [79,116]. Importantly, THR-β is intricately linked to other pathways of interest in NAFLD, including PPAR-α, FGF-21 and UCP1 [79,117,118]. Since THR-β is interrelated to many metabolic pathways, it has been the focus of both pharmacologic and non-pharmacologic therapies. Resmetirom is an emerging therapy in NASH clinical trials that has the potential to significantly reduce hepatic fat, making it a potential therapy for patients with NASH [14].

Regular physical activity, including exercise training, is also well known to significantly impact circulating levels of thyroid hormone at the population level [119], in smaller groups of healthy adults who perform regular resistance training [120] and also in post-menopausal women with metabolic syndrome [121]. Specifically, exercise training leads to increased turnover of T3 and T4 at the same work rate, effectively lowering resting concentrations of thyroid hormone. The intensity of exercise is an important consideration as it appears to cause differential effects on thyroid hormones, with vigorous intensity leading to the greatest change [122]. When limiting solely to NAFLD and NASH, the data are more sparse. While animal models of NASH have demonstrated that aerobic exercise training significantly reduced liver fat, inflammation and, more importantly, liver fibrosis progression [123], we are unaware of any studies in human populations exploring the differential impact of exercise training on thyroid receptors or thyroid hormone levels and would suggest this as an avenue ripe for future exploration given the interest in resmetirom as a NASH therapy which may become regulatory-agency-approved in the foreseeable future, and THR-β remains an additional target for which no concrete conclusions can be drawn in relationship to exercise training.

**Figure 1:**
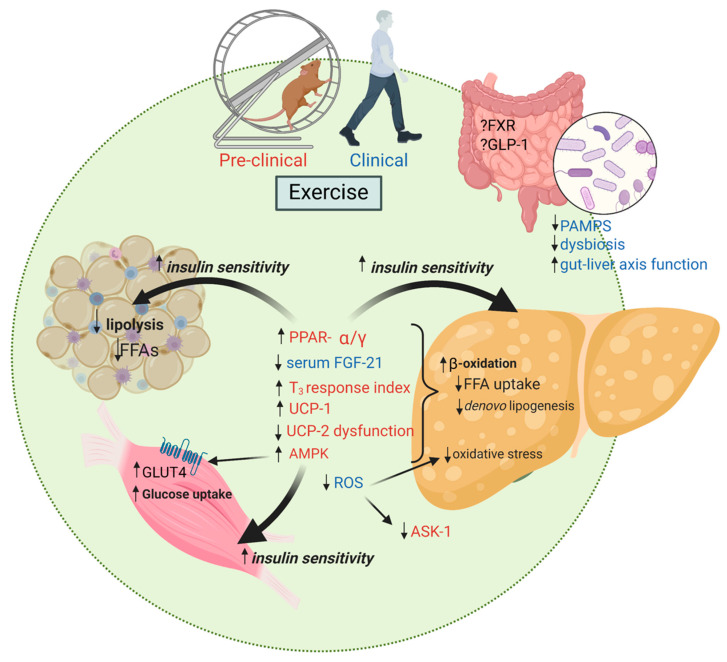
Exercise training favorably impacts multiple mechanisms of action important in the development of NAFLD and disease progression to NASH. FFAs, free fatty acids; PPAR-α/γ, peroxisome proliferator-activated receptor alpha/gamma; FGF-21, fibroblast growth factor-21; T_3_, triiodothyronine; UCP, mitochondrial uncoupling proteins; AMPK, adenosine monophosphate-activated protein kinase; ROS, reactive oxygen species; ASK, apoptosis signal-regulating kinase; FXR, farnesoid X receptor; GLP-1, glucagon-like peptide 1; PAMPS, pathogen-associated molecular patterns; GLUT-4, glucose transporter type 4. Created with BioRender.com.

Figure 1 summarizes the mechanistic pathways of interest in explaining why exercise can reduce hepatic steatosis, liver inflammation and liver fibrosis.

## 3. Exercise Training and Mechanistic Pathways Involved in the Development of Liver Inflammation and Fibrosis

### 3.1. Apoptosis Signal-Regulating Kinase 1 (ASK-1) and Endoplasmic Reticulum Stress (ERS)

Apoptosis is one of the final reactions that contributes to liver injury in NASH and is mediated through an important mitochondrial pathway involving the ASK-1 protein kinase. ASK-1 is known to regulate a complex set of tasks affected by ERS, and it plays a key role in hepatocyte injury, inflammation, and liver fibrosis. For these reasons, ASK-1 is an attractive molecular target in the treatment of NAFLD and NASH. Unfortunately, despite promising early-phase results, the STELLAR-3 and -4 trials failed to reach their primary endpoint and show improvement in liver fibrosis with the ASK-1 inhibitor selonsertib [124,125].

The relationship between exercise training and ASK-1 is not well explored. While animal models of obesity have demonstrated that regular aerobic exercise with swimming can attenuate insulin resistance through the regulation of ASK-1-mediated insulin signaling [126], animal models of NAFLD have not examined changes in ASK-1 signaling directly and have demonstrated inconsistent results with multiple studies showing both increased and decreased ERS in the liver [127]. For these reasons, no strong conclusions can be made about the relationship between exercise training, ASK-1 and ERS in NAFLD (Table 2).

### 3.2. Farnesoid X Receptor (FXR), Bile Acids and the Microbiome

FXR controls bile acid synthesis and can influence lipid and glucose homeostasis. It has been shown that mice without proper expression of FXR in the gut are prone to NAFLD, obesity and liver cancers [128,129,130]. Whether a result of altered gut microbiota or a change in expression of FXR, disruptions in the circulating bile acid levels contribute towards negative NAFLD-related outcomes and make for a potentially obvious target in both exercise and drug intervention. The FLINT Trial investigated the benefit of FXR agonist obeticholic acid and demonstrated a significant reduction in both hepatic fat and also liver fibrosis; however, owing largely to an unfavorable side effect profile, including high rates of pruritus, this medication did not achieve regulatory agency approval [131]. Newer-generation FXR agonists continue to be developed in the hopes of avoiding these limiting side effects while preserving efficacy, including tropifexor [9] and cilofexor [10], for which early-phase studies are quite promising.

While exercise training impacts bile acids and the FXR pathway, the results are inconsistent, and in some cases, FXR signaling is not impacted at all, at least in animal models [132,133]. In healthy individuals and high-level endurance athletes, the overall bile acid pool decreases in response to exercise training [134,135]. In patients with NAFLD, the impact of exercise training on bile acids and the FXR pathway remains largely unexplored, although a small pilot study demonstrated that aerobic exercise training reversed the dysbiosis that is the hallmark of NAFLD and NASH, which could potentially change the levels of secondary bile acids and affect the bile acid pool indirectly [136].

The gut microbiome is a complex place of residence for numerous organisms. While diet is often one of the primary factors for the health and composition of the gut microbiome, evidence is emerging to suggest exercise may have a similar role in microbiome health [137,138]. An altered gut microbiota is the cornerstone of gut–liver axis disruption in NAFLD [139,140]. Both physical inactivity as well as a high-fat, low-fiber diet has a direct effect on the gut microbiota composition through increased production of pathogen-associated molecular patterns (PAMPs), peptides, endogenous alcohols and decreased short-chain fatty acids, amongst other factors. Collectively, this leads to disruption of the gut mucosal barrier and increased intestinal permeability or “leaky gut”, which allows further interaction of the microbiota-produced PAMPs and metabolites with the mucosal surface immune cells [139,140]. Secondary bile acid levels are also increased with the gut–liver axis dysfunction seen in NAFLD. This reduces intestinal FXR signaling which disrupts the gut vascular barrier integrity, allowing delivery of PAMPs and metabolites via the portal vein to the lipid-laden liver which is already predisposed to further injury. In patients with NAFLD and NASH, several studies have shown the differential impact of exercise training on reversing dysbiosis, restoring a healthy gut–liver axis, which would be expected to lead to resolution of “leaky gut”, and offering potential through a reduction in the delivery of gut-derived pathogenic factors to ameliorate NAFLD and NASH [136,141,142].

In summary, while clinical trials are limited by sample size, exercise appears to impact the gut–liver axis in patients with NAFLD; whether this is mediated through FXR activation remains unclear.

### 3.3. Reactive Oxygen Species (ROS)

Oxidative stress is caused when an imbalance occurs between the production and accumulation of ROS, which ultimately leads to impaired ability to detoxify and remove these reactive products [143]. ROS play a key role in NAFLD development and disease progression to NASH through a myriad of complex mechanisms that contribute to cellular damage, inflammation, and fibrosis [144]. These include lipid peroxidation, mitochondrial dysfunction, inflammatory pathway activation and insulin resistance, which worsens hepatic steatosis and inflammation [145]. Additionally, ROS induce epigenetic modifications, disrupt bile acid homeostasis and affect the gut–liver axis, leading to liver inflammation and injury [146]. They interact with other reactive species, amplifying oxidative stress and cellular damage, and modulate cellular signaling, resulting in hepatocyte dysfunction and liver injury [147]. Furthermore, ROS disrupt cellular calcium homeostasis, influence immune cell function and impact extracellular matrix remodeling, contributing to liver fibrosis and NAFLD progression [148]. ROS also regulate microRNA expression and modulate macrophage polarization, affecting inflammation and fibrosis in NAFLD [149].

Excess lipid accumulation is believed to impair the electron transport chain, ultimately leading to a direct and unintended interaction between electrons and oxygen, which creates ROS [150]. CYP2E1 is also a direct source of ROS in NAFLD and NASH. Vitamin E, an antioxidant known to prevent lipid peroxidation, scavenge ROS and protect cellular membranes from oxidative damage, can combat the effects of ROS in patients with NASH. In fact, the landmark PIVENS Trial [96] demonstrated that vitamin E can significantly reduce hepatic steatosis. More recent evidence suggests that vitamin E can also reverse liver fibrosis and decrease major adverse liver outcomes, improving transplant-free survival in patients with advanced NASH [151].

Exercise training has been shown to reduce serum oxidative stress levels without the need for dietary restriction in patients with obesity [152]. Exercise training suppresses ROS overproduction by upregulating several essential antioxidant enzymes and anti-inflammatory mediators in NAFLD [109]. The nuclear factor E2-related factor (Nrf2), a transcription factor that plays a key role in the upregulation of endogenous antioxidant defenses, is crucial in regulating the body’s antioxidant response, defending against oxidative stress [153], and can be activated by exercise training in human skeletal muscle [154]. Exercise training can activate AMPK signal pathways, resulting in increased ROS production in the mitochondria due to higher oxygen consumption and metabolic rate. This transient increase in ROS levels activates the Nrf2 pathway, which in turn upregulates the expression of antioxidant genes, such as heme oxygenase-1, catalase, NAD(P)H quinone oxidoreductase 1 and glutamate-cysteine ligase [155]. These enzymes help neutralize ROS, mitigate oxidative damage, and maintain cellular redox homeostasis. The activation of the Nrf2 pathway enhances the antioxidant capacity, reduces inflammation and improves insulin sensitivity, alleviating hepatic steatosis and inflammation and slowing NAFLD progression [153]. However, whether the activation of Nrf2 by exercise is observed in patients with NAFLD and NASH as a mechanism underlying how exercise training reverses ROS production and damage needs further investigation. Moreover, activation of signaling pathways such as AMPK and peroxisome proliferator-activated receptor-γ coactivator-1α due to exercise training triggers liver mitochondrial biogenesis which then activates the antioxidant system, concomitantly decreasing the levels of oxidative stress [61]. Protein synthesis necessary for cellular function and repair, including antioxidant enzymes, is facilitated through increased nutrient delivery during exercise [156]. Increased oxygen delivery due to improved blood flow to the liver during exercise can further enhance the production of antioxidant enzymes. Additionally, autophagy in hepatocytes is activated during exercise training, thus removing damaged mitochondria, improving mitochondrial function, reducing oxidative stress and promoting ROS abolition [157].

## 4. Conclusions

As rates of NAFLD and NASH increase worldwide in parallel with the obesity pandemic, there are key unmet needs to better understand this complicated disease. Despite years of scientific effort, a regulatory-agency-approved treatment for NAFLD and NASH is not yet available, although there are several promising medications on the horizon. Even when these medications are approved, regular physical activity, which is most effectively accomplished through a formal and supported exercise training program, will continue to be a vital component in not only the clinical management of NAFLD but also in its prevention. Moreover, emerging evidence suggests that exercise training at levels promoted in population physical activity guidelines is just as efficacious in MRI-measured liver fat reduction as anti-steatogenic medications which are both prescribed off-label and under development [8]. The question of the synergistic effect of exercise training with various drugs under development remains unknown. It is plausible that exercise training impacts nearly every therapeutic target of interest in NASH drug discovery. We look to future research to not only better define the mechanisms underlying the benefit of regular physical activity and exercise training in patients with NAFLD and NASH but also to further our exploration of the epigenetic-related impact of exercise training and precision-medicine-based therapeutic response. With this future understanding, we can not only impact the one out of three adults with NAFLD worldwide but also the billions of individuals living with other chronic metabolic diseases.

## Figures and Tables

**Table 1:** Selected mechanisms of action of exercise on hepatic steatosis.

Mechanism of Action	Effects on Hepatic Steatosis	Animal Evidence	Human Evidence	Extrahepatic Benefits	Safety
AMPK	↓ steatosis	Aerobic and resistance training increases AMPK activation	N.A.	↑ GLUT-4 expression	Exercise is safe for people with NAFLD/NASH when supervised by an appropriately qualified exercise professional and with adequate screening and monitoring protocols.
				↑ glucose uptake	
FGF-19 and -21	↓ steatosis	Aerobic exercise training increases FGF receptor 1 and FGF receptor 2 Chronic exercise training increases β-klotho Chronic exercise training reduces serum FGF-21	Aerobic and resistance training reduces serum FGF-21	↑ hepatic glycogen synthesis	
			Effect of exercise on FGF-19 is unclear	↑ hepatic gluconeogenesis	
GLP-1	↓ steatosis	N.A.	Acute aerobic exercise ↑ GLP-1	↑ weight loss	
			Short term high-intensity aerobic exercise reversed GLP-1 resistance	↑ insulin production	
			N.A. for chronic training adaptation	↓ appetite	
Mitochondrial function and β-oxidation	↓ steatosis	Exercise training improves mitochondrial function, ↑ mitochondrial content and ↑ β-oxidation	Exercise training improves mitochondrial function, ↑ mitochondrial content and ↑ β-oxidation	↑ insulin sensitivity	
UCP	↓ steatosis	Aerobic exercise upregulates UCP-1 and reverses UCP-2 dysfunction in the liver	N.A.	Prevent oxidative stress	
PPAR-α/γ	↓ steatosis	Exercise training (moderate-intensity running or swimming; high-intensity interval training; resistance training) activates PPAR-α	N.A.	↑ insulin sensitivity	
		Maternal aerobic exercise may protect against early life NAFLD in offspring			
THR-β	↓ steatosis	Aerobic exercise training increases T3 response index	N.A.	Acute exercise ↑ free T3 and T4	
				↑ mitochondria size and number	
				↑ glucose uptake	
				↑ gluconeogenesis	
				Chronic exercise ↑ T3 and T4 turnover at same absolute intensity	

AMPK = adenosine monophosphate-activated protein kinase; FGF = fibroblast growth factor; GLP = glucagon-like peptide; GLUT = glucose transporter type; N.A. = not available; NAFLD = nonalcoholic fatty liver disease; NASH = nonalcoholic steatohepatitis; PPAR = peroxisome proliferator-activated receptor; T3 = triiodothyronine; T4 = thyroxine; THR = thyroid hormone receptor; UCP = mitochondrial uncoupling proteins.

**Table 2:** Selected mechanisms of action of exercise on liver fibrosis.

Mechanism of Action	Effects on Liver Fibrosis	Animal Evidence	Human Evidence	Extrahepatic Benefits	Safety
ASK-1	↓ fibrosis	Aerobic exercise (swimming) regulates ASK-1-mediated insulin signaling in animal models of obesity	N.A.	↓ ERS	Exercise is safe for people with NAFLD/NASH when supervised by an appropriately qualified exercise professional and with adequate screening and monitoring protocols.
	↓ hepatocyte injury	No evidence in NAFLD/NASH			
FXR	↓ fibrosis	N.A.	N.A.		
ROS	↓ fibrosis via a reduction in inflammation	N.A.	Aerobic exercise reduces ROS		

ASK = apoptosis signal-regulating kinase; ERS = endoplasmic reticulum stress; FXR = Farnesoid X receptor; N.A. = not available; NAFLD = nonalcoholic fatty liver disease; NASH = nonalcoholic steatohepatitis; ROS = reactive oxygen species.

## Abbreviations

Adenosine monophosphate-activated protein kinase, AMPK; Apoptosis signal-regulating kinase 1, ASK-1; endoplasmic reticulum stress, ERS; Farnesoid X receptor, FXR; FGF, fibroblast growth factor; glucagon-like peptide, GLP; high-intensity interval training, HIIT; NASH, nonalcoholic steatohepatitis; NAFLD, nonalcoholic fatty liver disease; nuclear factor E2-related factor, Nrf2; pathogen-associated molecular patterns, PAMP; peroxisome proliferator-activated receptor, PPAR; reactive oxygen species, ROS; thyroid hormone receptor, THR; uncoupling proteins, UCP.
